# Supplementary material for: Young women’s reproductive health conversations: Roles of maternal figures and clinical practices
Source: PLoS One. 2020 Jan 23;15(1):e0228142. doi: 10.1371/journal.pone.0228142 (PMC6977719; doi:10.1371/journal.pone.0228142)
Supplement: Attachment — Submitted filename: PLOS Review.docx [file pone.0228142.s002.docx]

- Is it possible to put the total number of focus group participants in the abstract?
- Should the concept of paternalism be added to the abstract since it’s a major part of this manuscript?
- Lines 80-87: Why isn’t there a mention about the specific role of father’s? If the focus of the manuscript is mothers over fathers, it should be explained why.
- Line 106: Is parental authority always an imposition?
- Lines 109-110: “We focus specifically on mothers’ imposition in the reproductive health decisions of their daughters. “ Is this statement too harsh? Are you exploring whether this happens or how it happens? Or are you saying it happens all the time?
- Lines 93-110: Could you explain more about the theme of paternalism that you found in your primary analysis of this data?
  - Is paternalism always negative?
  - Could you define medical paternalism?
  - What is the difference between parental paternalism and medical paternalism?
  - How can an adolescent be protected from both parental paternalism and medical paternalism to ensure they are making their own autonomous decision? Or is it even possible? This could possibly be included in the discussion section.
    - Or is medical paternalism important to this manuscript since it was not mentioned in the discussion/conclusion sections?
  - How does amplifying the role of a parent, help against medical paternalism?
  - Could you explain more about paternalism and reproductive health decision-making? Is there a difference between parental paternalism versus medical paternalism in reproductive decision-making?
  - It might be helpful to include how paternalism can hinder (or may help?) the ability of young women to have confidential conversations with their provider.
- Line 112: Could you explain more about the role of the CAB for your study beyond what is stated in Line 151-154? Why was there interpretation of the data important (Lines 259-274)?
- Lines 121-122: “Hispanic women could choose a focus group with women of similar race or ethnicity.” Why was this decided?  Why couldn’t other race of women do the same?
- Table 1: Could you add the racial breakdown of the final two focus groups (Aged 18-28) and the average age of these focus groups?
- Discussion/Conclusion sections: From the results sections it appears that some of the young women did not mind or wanted their mothers to be in the room in their discussions with their providers. In lines 299-201 and lines 319-320, you discussed that health care providers would like to provide oral contraceptives to young women without their parent’s consent and you stated that young women and providers benefit from situations where the provider asks the parent to leave the room. However how should it be navigated when the young woman wants her mother involved?
  - Additionally shouldn’t we still have concerns about medical paternalism? For example, if a young woman comes into the clinic and wants birth control pills, but through private counseling with the provider she is convinced to get an IUD, are we sure she made an autonomous decision?
  - How do we ensure a young woman makes an autonomous decision about her reproductive health whether the mother is in the clinic room or not?
